# A Comparative Analysis of Transcription Networks Active in Juvenile and Mature Wood in *Populus*

**DOI:** 10.3389/fpls.2021.675075

**Published:** 2021-05-28

**Authors:** Laifu Luo, Yingying Zhu, Jinshan Gui, Tongmin Yin, Wenchun Luo, Jianquan Liu, Laigeng Li

**Affiliations:** ^1^State Key Laboratory of Grassland Agro-Ecosystem, School of Life Sciences, Lanzhou University, Lanzhou, China; ^2^National Key Laboratory of Plant Molecular Genetics, CAS Center for Excellence in Molecular Plant Sciences, Institute of Plant Physiology and Ecology, Chinese Academy of Sciences, Shanghai, China; ^3^College of Forestry, Nanjing Forestry University, Nanjing, China

**Keywords:** juvenile wood, mature wood, wood property, wood formation, RNA-seq, cell wall, cell expansion, DNA methylation

## Abstract

Juvenile wood (JW) and mature wood (MW) have distinct physical and chemical characters, resulting from wood formation at different development phases over tree lifespan. However, the regulatory mechanisms that distinguish or modulate the characteristics of JW and MW in relation to each other have not been mapped. In this study, by employing the *Populus* trees with an identical genetic background, we carried out RNA sequencing (RNA-seq) and whole genome bisulfite sequencing (WGBS) in JW and MW forming tissue and analyzed the transcriptional programs in association with the wood formation in different phrases. JW and MW of *Populus* displayed different wood properties, including higher content of cellulose and hemicelluloses, less lignin, and longer and larger fiber cells and vessel elements in MW as compared with JW. Significant differences in transcriptional programs and patterns of DNA methylation were detected between JW and MW. The differences were concentrated in gene networks involved in regulating hormonal signaling pathways responsible for auxin distribution and brassinosteroids biosynthesis as well as genes active in regulating cell expansion and secondary cell wall biosynthesis. An observed correlation between gene expression profiling and DNA methylation indicated that DNA methylation affected expression of the genes related to auxin distribution and brassinosteroids signal transduction, cell expansion in JW, and MW formation. The results suggest that auxin distribution, brassinosteroids biosynthesis, and signaling be the critical molecular modules in formation of JW and MW. DNA methylation plays a role in formatting the molecular modules which contribute to the transcriptional programs of wood formation in different development phases. The study sheds light into better understanding of the molecular networks underlying regulation of wood properties which would be informative for genetic manipulation for improvement of wood formation.

## Introduction

Perennial woody plants are characterized by large size and a long lifespan, in which a long non-flowering period of juvenile phase can last years to decades, for example, 3–5 years in *Populus* and 10–15 years in *Pinus* (Braatne et al., 1996; Owens, 2006). Wood produced during juvenile phase is called juvenile wood (JW) which is followed by a mature phase during which trees start flowering and producing mature wood (MW) outside of JW (Basheer-Salimia, 2007). Compared with JW, MW is characterized with longer xylem cells, thicker secondary cell walls, lower density of vessels, higher crystallinity of cellulose in fibers, and smaller microfibril angles (Barrios et al., 2017). Thus, MW is more desirable from a processing and utilization perspective for construction wood, wood pulping, and fiber material production. As a matter of fact, to meet the increasing demand for raw wood material, artificial forest plantation aims to reduce the rotation length and enhance productivity, which makes JW with lower wood quality as a major source for wood industry (Moore and Cown, 2017). This seriously affects the utilization and processing of wood. How to make wood to mature quickly and the proportion of JW to be reduced has become an important aspect of improving wood properties.

Wood formation starts with cell divisions at vascular cambium and subsequent differentiation into secondary xylem through cell expansion, secondary cell wall thickening, and programmed cell death (Fromm, 2013). Plant hormones, such as auxin, brassinosteroids, and gibberellin, participate in regulation of wood formation (Israelsson et al., 2005; Demura and Fukuda, 2007; Choi et al., 2017). The size of wood cells depend on cell expansion process while mechanical and chemical properties of wood are largely determined by secondary cell wall thickening (Cosgrove, 2018). Cell expansion is controlled by extension of the primary cell wall, which is composed of 20–30% cellulose, 30–50% pectins, 20–25% hemicelluloses, and 10% glycoproteins (Mcneil et al., 1984). Following cell expansion, wall thickening is initiated with transcriptional programs for secondary cell wall biosynthesis (Plomion et al., 2001). Secondary cell walls are composed of 40–80% cellulose, 10–40% hemicellulose, 5–25% lignin, and glycoproteins (Kumar et al., 2016). As JW and MW display distinct wood properties, likely the secondary cell formation in JW and MW is differentially regulated.

DNA methylation, a critical epigenetic mechanism among eukaryotes, affects many biological processes. In plant, most of DNA methylation occurs at the fifth carbon of cytosine (including three cytosine contexts, CG, CHG, and CHH, where H represents A, C, or T) to form 5-methylcytosine by DNA methyltransferase (Goll and Bestor, 2005; Law and Jacobsen, 2010; He et al., 2011). Evidence indicates that DNA methylation can regulate gene expression in numerous biological processes including response to abiotic stresses (Wang et al., 2011; Dowen et al., 2012; Ci et al., 2015; Su et al., 2018; Liang et al., 2019), plant development and morphogenesis (Lafon-Placette et al., 2013), and wood formation (Wang et al., 2016). The degree of DNA methylation is also related to plant development phases. The degree of DNA methylation at mature phase was significantly higher than that at juvenile phase in *Pinus radiate* (Fraga et al., 2002a). DNA methylation increases along with the age extension in some species (Fraga et al., 2002b). It is unclear how DNA methylation is involved in regulation of JW and MW formation.

Despite studies which have shown physicochemical difference of wood properties between JW and MW, the molecular regulatory networks underlying formation of the different wood properties is not fully elucidated. In this study, by employing *Populus* trees with an identical genetic background, we analyzed different physical and chemical characters in association with the transcriptomic profiles and DNA methylation during the formation of JW and MW. Correlation analysis revealed the transcriptional networks and DNA methylation that are involved in regulation of wood formation with different wood properties. This study provides an array of mechanistic information for understanding of JW and MW formation, as well as new clues for genetic manipulation for improvement of wood properties.

## Materials and Methods

### Tissue Sampling

*Populus* trees propagated from the same clone (*Populus deltoides* × *P. euramericana* cv. “*Nanlin895*”) were grown in the same plantation located at Siyang, Suqian, Jiangsu, China (33° 47′ N, 118° 22′ E). Wood-forming tissues were sampled from 1-m trunk above the breast height (1.3 m from ground) from 2-year-old (formation of JW) and 8-year-old trees (formation of MW) at fast growing time (May 2017). After bark was removed, wood-forming tissue (developing secondary xylem) was collected directly into liquid nitrogen and stored at −80°C freezer for later analysis (Song et al., 2011). Three trees as biological replicates were sampled (Supplementary Figure 1), respectively.

### Analysis of Wood Properties

After developing xylem was collected, the tree trunk was used for wood analysis. JW and MW were sampled as illustrated in Supplementary Figure 1. Wood tissue was sectioned into 20 μm in thickness and stained with 0.5% phloroglucinol in 12% HCl. Cross sections were observed under a microscope (Olympus, BX53). The number of fibers and vessels and their cross area were counted using Image J. Meanwhile, the wood cells were separated after treatment using acetic acid/hydrogen peroxide (1:1, *v*/*v*) solution at 80°C for 6 h. The separated wood cells were then stained with safranine (1% in water), and the length of fiber cells and vessels was measured under a microscope (Olympus, BX53) using Image J.

### Cell Wall Composition Determination

Air-dried wood sample was ground into powder and filtered through 60-mesh sieve. According to our previous established protocol (Yu et al., 2014), alcohol-insoluble residues (AIR) were firstly obtained by extracting the wood powder with 70% ethanol, chloroform/methanol (1:1, *v*/*v*), and acetone. Amylase and pullulanase in 0.1 M sodium acetate buffer (pH 5.0) were used to treat the extracted AIR overnight. For analysis of the sugar in hemicelluloses, AIR was treated with 2 M trifluoroacetic acid (TFA) at 121°C for 90 min. The supernatant was evaporated and incubated in 20 mg/ml fresh sodium borohydride solution at 40° for 90 min. The product was then neutralized with acetic acid and mixed with 1-methylimidazole and acetic anhydride for acetylation. After extraction with dichloromethane, the product was mixed with ethyl acetate for GC-MS (6890N GC system and 5975 Mass detector, Agilent Technologies, equipped with a SP-2380 capillary column, Supelco, Sigma-Aldrich) analysis. Meanwhile, standard sugars were used to calibrate sugar content determined in samples. The insoluble precipitate from the AIR treated with TFA was collected for crystalline cellulose content determination. The Updegraff reagent (acetic acid:nitric acid:water, 8:1:2, *v*/*v*) was added to the precipitate and incubated at 100°C for 30 min. After washing with H_2_O and acetone, the precipitate was incubated with 72% sulfuric acid at room temperature for 1 h. The content of crystalline cellulose was determined by anthrone assay (Foster et al., 2010b). For lignin measurement, AIR was incubated with freshly prepared acetyl bromide (25%, acetyl bromide in acetic acid) at 50°C for 3 h. After cooling, the AIR was mixed with 2 M NaOH, 0.5 M fresh hydroxylamine hydrochloride, and acetic acid. Lignin content was determined using a microplate reader (Varioskan Flash, Thermo) (Foster et al., 2010a).

### RNA Isolation and RNA Sequencing

Total RNA was extracted from wood-forming tissues using a mirVana miRNA Isolation Kit (Ambion-1561) following the manufacturer's instruction. After being treated by RNase-free DNase I (Sigma, 4716728001), the quality of total RNA was assessed on NanoDrop spectrophotometer (NanoDrop 2000, Thermo Scientific) and on agarose gel electrophoresis. For RNA sequencing (RNA-seq), cDNA library was generated from 5 μg of total RNA with TruSeq Stranded mRNA LTSample Prep Kit (Illumina, RS-122-2101) and Agencourt AMPure XP (BECKMAN COULTER, A63881). cDNA library was qualified through length distribution of fragments using Agilent 2100 (Bioanalyzer). The 150-bp paired-end sequencing was performed using platform of Illumina HiSeq X10. About five million reads per samples were generated.

### DNA Isolation and Bisulfite Sequencing

Genomic DNA was extracted from wood-forming tissues using QIAamp DNA Mini kit (Cat.51306, Qiagen). DNA quantification and integrity were determined by a Nanodrop spectrophotometer (Thermo Fisher Scientific, Inc., Wilmington, DE) and 1% agarose electrophoresis, respectively. Before bisulfite treatment, lambda DNA was added to the purified DNA, which was used as an internal reference to calculate the conversion rate. The mixed DNA was then bisulfite treated using a Zymo Research EZ DNA methylaiton-Glod Kit (Zymo, D05005). Bisulfite sequencing (BS-seq) libraries were constructed by TruSeq® DNA Methylation Kit (Illumina, EGMK91396) following the manufacturer's instruction. After libraries were qualified, sequencing was performed on the Illumina HiSeq X Ten platform and 150 bp paired-end reads were generated.

### Analysis of Transcriptome Sequencing Data

Raw reads of sequencing were processed using NGS QC Toolkit to remove low-quality reads (Patel and Jain, 2012). The cleaned reads were mapped to *Populus trichocarpa*'s genome (http://phytozome.jgi.doe.gov/) using hisat2 with default parameters (Kim et al., 2015). Gene expression level was measured as fragments per kilobase per million reads (FPKM) using cufflinks (Trapnell et al., 2010; Roberts et al., 2011). Read counts for each gene in each sample were obtained using htseq-count and standardized by rlog (Anders et al., 2015). Principle component analysis (PCA) was performed by plotPCA of DEseq2 R package with default parameters. Differential expression genes (DEGs) were identified using the DESeq R package by estimation of Size Factors and nbinomTest. Analysis of DEGs with gene ontology (GO) enrichment and the Kyoto Encyclopedia of Genes and Genomes (KEGG) (Kanehisa et al., 2008) pathway enrichment was performed using R based on the hypergeometric distribution.

### Analysis of Genome Bisulfite Sequencing

The raw reads of BS-seq were cleaned using Fastp (Chen et al., 2018) by removing adapters, ploy-N, and low-quality reads. The remaining high-quality clean reads were mapped to the *Populus trichocarpa*'s genome (http://phytozome.jgi.doe.gov/) using Bismark software with default parameters (Krueger and Andrews, 2011). Methylcytosine (mC) sites were identified using MethylKit (Akalin et al., 2012). With default parameters, MethylKit was applied for PCA analysis. Differentially methylated regions (DMRs) were identified using MethylKit software with a *Q* value (*p*-value corrected by FDR method) threshold of 0.05 and an absolute delta cutoff of 10% between the two groups. Analysis of DMGs with GO enrichment and KEGG pathway enrichment was performed according to the same method used for DEGs analysis.

### Quantitative Real-Time PCR

The first-strand cDNA was synthesized from 2 μg of total RNA using a cDNA Synthesis SuperMix (TransGen Biotech, AT311-03). Using cDNA as template, quantitative real-time PCR (qRT-PCR) was performed using *Perfectstart*™ Green qPCR SuperMix (TransGen, AQ601) and a Quantstudio™ 3 Real-Time PCR Detection System (Thermo). The primers used for selected genes are listed in Supplementary Table 13, and *TUB9* was used as an internal control to normalize gene expression.

## Results

### Properties of JW and MW in *Populus*

**Figure 1:**
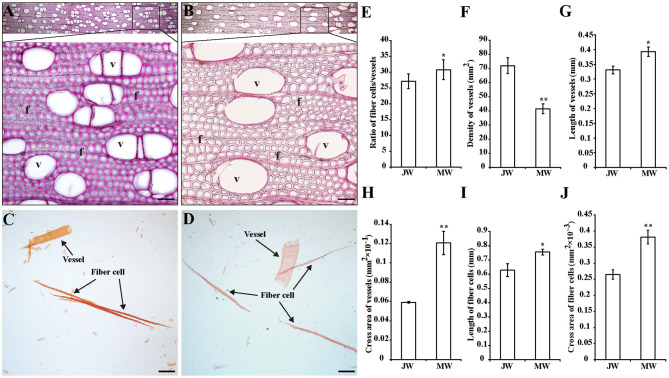
Properties of the wood produced in juvenile phase (JP) and mature phase (MP). **(A,B)** Wood sections stained with phloroglucinol in different magnifications from juvenile wood (JW) **(A)** and mature wood (MW) **(B)**. Bars = 50 μm. **(C,D)** Fibers and vessels from JW **(C)** and MW **(D)**. Bars = 200 μm. **(E)** Ratio of numbers of fiber cells/vessels. **(F)** Density of vessels. **(G,H)** Length and cross area of vessels. The values were means ± SE of 500 and 800 independent vessels from JW and MW, respectively. **(I,J)** Length and cross area of fiber cells. The values were means ± SE of 1,000 independent fibers from JW and MW, respectively. Significance was determined by Student's *t*-test (*p < 0.05 and **p < 0.01). f, fiber cell; v, vessel.

To examine the properties of the JW and MW produced in *Populus*, plantation-grown trees that were propagated from a single clone were sampled. Three trees at 2 and 8 years old were collected with trunk at breast height, respectively (Supplementary Figure 1). In wood anatomical section, difference in the ratio of fiber cell/vessel, the length and size of fibers and vessels was observed between JW and MW (Figures 1A–D). MW contained higher ratio of fiber cell/vessel, lower density of vessel cell in wood section, and longer and larger fiber cell and vessel than those in JW (Figures 1E–J). Chemical analysis indicated that MW contained higher content of crystalline cellulose and lower content of lignin compared with JW (Table 1). Sugar composition in hemicelluloses also showed difference between JW and MW. MW contained higher xylose, mannose, glucose, and arabinose but lower galactose compared with JW (Table 1). These results indicated that JW and MW in *Populus* displayed different cellular structures and chemical compositions.

**Table 1:** Chemical composition in JW and MW of *Populus*.

**Chemical composition (μg/mg AIR)**	**JW**	**MW**
Cellulose	400.3 ± 24.4	432.4 ± 27.0^*^
Lignin	223.1 ± 9.7	206.7 ± 18.9^*^
**Hemicellulose**	
Xylose	145.9 ± 14.8	192.4 ± 18.1^**^
Mannose	27.2 ± 4.7	36.8 ± 4.2^**^
Galactose	2.7 ± 0.5	1.8 ± 0.33^*^
Glucose	62.4 ± 6.5	69.8 ± 6.0^*^
Arabinose	2.5 ± 0.2	3.6 ± 0.3^**^

*Sugar content in hemicelluloses is calculated on the basis of standard sugar calibration. Significance was determined by Student's t-test (*

* *p < 0.05 and*

** *p < 0.01)*.

### Transcriptional Profiles in Formation of JW and MW

To dissect the gene expression involved in *Populus* wood formation, transcripts were profiled in the wood-forming tissues undergoing formation of JW and MW *via* high-throughput RNA-seq. Assessment of the RNA-seq data and biological repeats validated the high quality of the sequence data generated from the wood-forming tissues (Supplementary Table 1, The raw data in Sequence Read Archive (SRA), ID: PRJNA705066). A total of 300.5 million raw reads were obtained from six samples, and about 284.8 million high-quality reads (more than 94% of raw reads) were obtained after filtering and removal of low-quality reads. More than 86% of the high-quality reads per sample were mapped to the reference genome, corresponding to expression of ~20,000 genes out of 41,335 predicted genes in each sample. Meanwhile, PCA indicated that the transcript profiles showed a clear separation between JW and MW (Supplementary Figure 2A), suggesting the different transcription activities in formation of JW and MW.

**Figure 2:**
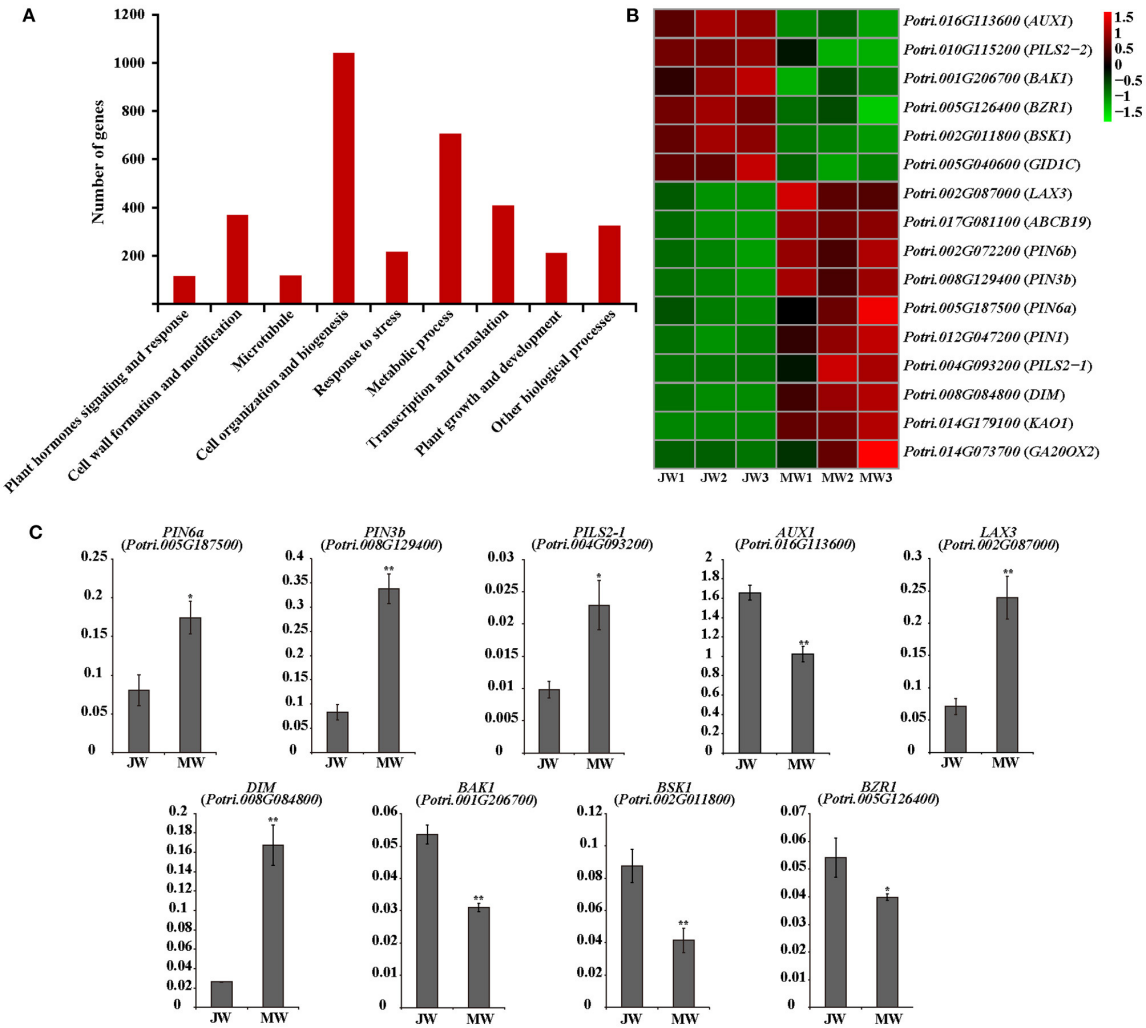
**(A)** The functional of the differentially expressed genes (DEGs). **(B)** The heat map of genes related to plant hormones. **(C)** The results of quantitative real-time PCR of selected genes related to plant hormones in JW and MW. Significance was determined by Student's *t*-test (*p < 0.05 and **p < 0.01).

A group of 3,992 genes were identified (FPKM ≥3, fold change >2, and *p* value FDR < 0.05) for their differential expression in JW and MW (Supplementary Figure 2B; Supplementary Table 2). The DEGs included 2,110 higher expression in JW and 1,882 higher expression in MW. As indicated by GO and KEGG enrichment analysis, the DEGs were primarily associated with plant hormones signaling and response, cell wall formation and modification, microtubule, cell organization and biogenesis, transcription, and other biological processes (Figure 2A; Supplementary Tables 3, 4).

### Expression of Hormone-Related Genes in Wood Formation at Different Phases

Among the detected DEGs were included ample hormone-related genes. Particularly, genes of auxin transportation, brassinosteroids (BR) biosynthesis, and signaling were identified for their remarkable difference of expression in JW and MW (Figure 2B). The homologs of *AUX1, LAX3, PILS2*, and *ABCB19* which are related to auxin transport (Enders and Strader, 2015) had high expression level in JW and MW (FPKM >100). Intriguingly, the homolog (*Potri.016G113600*) of *AUX1* which facilitates auxin influx was expressed in JW higher than in MW, while the homolog (*Potri.017G081100*) of *ABCB19* which facilitates the efflux of auxin was expressed in MW higher than in JW. Furthermore, four homologs of *PIN1, PIN3*, and *PIN6*, encoded the auxin transporters that mediate that auxin efflux (Liu et al., 2014; Enders and Strader, 2015), were identified in DEGs, and their expression in MW was much higher than in JW (Figure 2B). In addition, the PIN-LIKES (PILS), which are thought to be located on the endoplasmic reticulum (ER), may transport auxin from the cytoplasm into the ER (Enders and Strader, 2015). One *PILS2* homolog (*Potri.004G093200*) was expressed in MW 13 times higher than in JW (Figure 2B). The differential expression of the auxin-related genes was verified by qRT-PCR determination (Figure 2C). These data indicate that the genes involved in IAA transport were differentially expressed in MW and JW.

Genes involved in BR biosynthesis and signaling were readily noticed among DEGs. The homolog (*Potri.008G084800*) of *DIM*/*DWF1* which is a key gene for BR biosynthesis in *Arabidopsis* (Klahre et al., 1998; Youn et al., 2018) was expressed in MW 5.6 times higher than in JW (Figure 2B). Additionally, several genes involved in BR signaling were differentially expressed between JW and MW (Figure 2B). The homolog of *BRI1-associated receptor kinase* (*BAK1*) (*Potri.001G206700*), *brassinosteroid signaling positive regulator* (*BZR1*) (*Potri.005G126400*), and *BR signaling kinase 1* (*BSK1*) (*Potri.002G011800*) were downregulated in MW compared with JW. qRT-PCR determination confirmed the differential expression (Figure 2C). On the other hand, although the genes involved in other hormones signaling such as gibberellins (GAs) were detected to be differentially expressed between JW and MW (Figure 2B), their expression profiles were unable to be verified. Together, the results suggest that auxin and BR may be involved in the regulation of JW and MW formation.

### Expression of Transcriptional Factor Genes in the Formation of JW and MW

**Figure 3:**
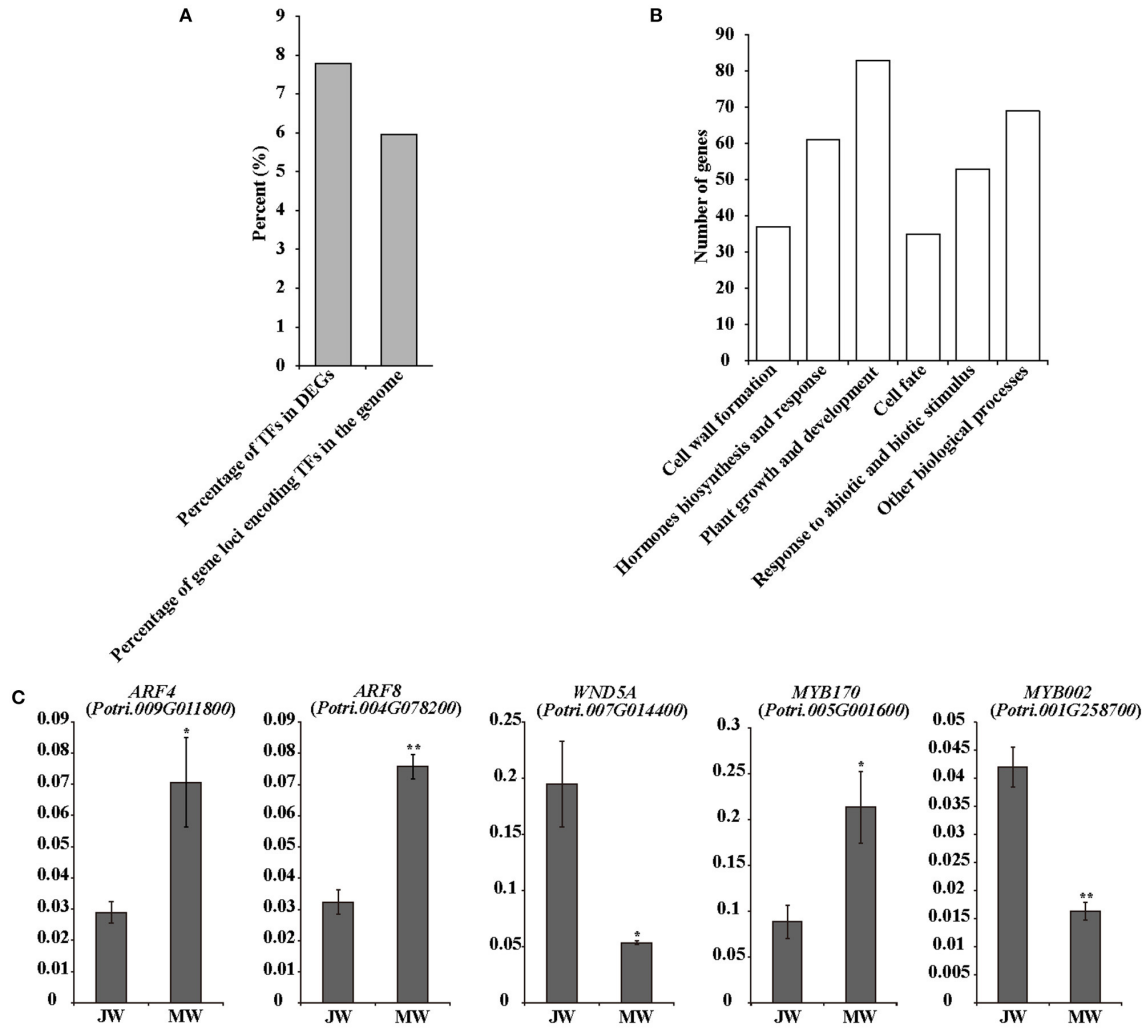
Transcriptional factors in DEGs. **(A)** Number of TF genes in detected DEGs. **(B)** Functions of the TFs among DEGs. **(C)** The results of quantitative real-time PCR of selected TFs in JW and MW. Significance was determined by Student's *t*-test (*p < 0.05 and **p < 0.01).

The identified 3,992 DEGs included 305 transcription factor (TF) genes, 7.6% of all DEGs, which is higher than 6% of TF genes in *Populus* genome (PlantTFDB, http://planttfdb.cbi.pku.edu.cn) (Jin et al., 2014) (Figure 3A; Supplementary Table 5), implying that expression of transcription factor genes is altered in higher proportion. Among the TF genes, 105 TF genes were upregulated in MW, and the rest were downregulated in MW. GO annotation analysis indicated that the TFs in the DEGs were primarily associated with hormone biosynthesis and responses, plant growth and development, cell fate, cell wall formation, and response to abiotic and biotic stimuli (Figure 3B).

Several TF genes that are involved in auxin signaling were differentially expressed in JW and MW (Supplementary Table 5). The DEGs contained nine *auxin response factor* (*ARF*) genes with four upregulated and five downregulated in JW. In addition, three homologs of *SHI-RELATED SEQUENCEs* (*SRSs*) that play a role in activation of auxin biosynthesis (Eklund et al., 2010) were upregulated in MW. One homolog of *AINTEGUMENTA-LIKE 6* (*AIL6*) that is involved in regulation of auxin biosynthesis (Pinon et al., 2013) displayed higher expression in MW. The differential expression determined by RNA-seq and qRT-PCR analyses was well-correlated (Figure 3C; Supplementary Figure 3).

The DEGs included a number of TFs related to regulation of cell wall formation, such as the homologs of *SND1, NST1 VND1*, and *VND4* (Supplementary Table 5), which were key TFs for regulation of secondary cell wall biosynthesis (Zhong et al., 2010; Hussey et al., 2013; Kumar et al., 2016). *PtrMYB26, PtrMYB90*, and *PtrMYB152* which were reported to directly regulate lignin biosynthesis in *Populus* (Zhong et al., 2011; Wang et al., 2014; Li et al., 2015) were expressed higher in JW than in MW. This is in agreement with the higher lignin content in JW. Furthermore, expression of *PtrMYB170* and *PtrMYB121*, which may play a role in resource acquisition and allocation for xylem development (Romano et al., 2012), were upregulated in MW. One homolog (*Potri.006G241700*) of *MYB3R1*, which played a role in activating expression of the genes in cell cycle (Haga et al., 2011), showed higher expression in MW. *PtrERF118* (*Potri.018G028000*), which was identified as a TF-regulating xylem cell expansion in *Populus* (Vahala et al., 2013; Seyfferth et al., 2018), was upregulated in MW. These results suggest that the transcriptional networks in relation to auxin biosynthesis and signaling, secondary cell wall formation, and xylem cell differentiation were differentially regulated in the formation of JW and MW.

### Expression of the Cell Wall Formation Genes in JW and MW

**Figure 4:**
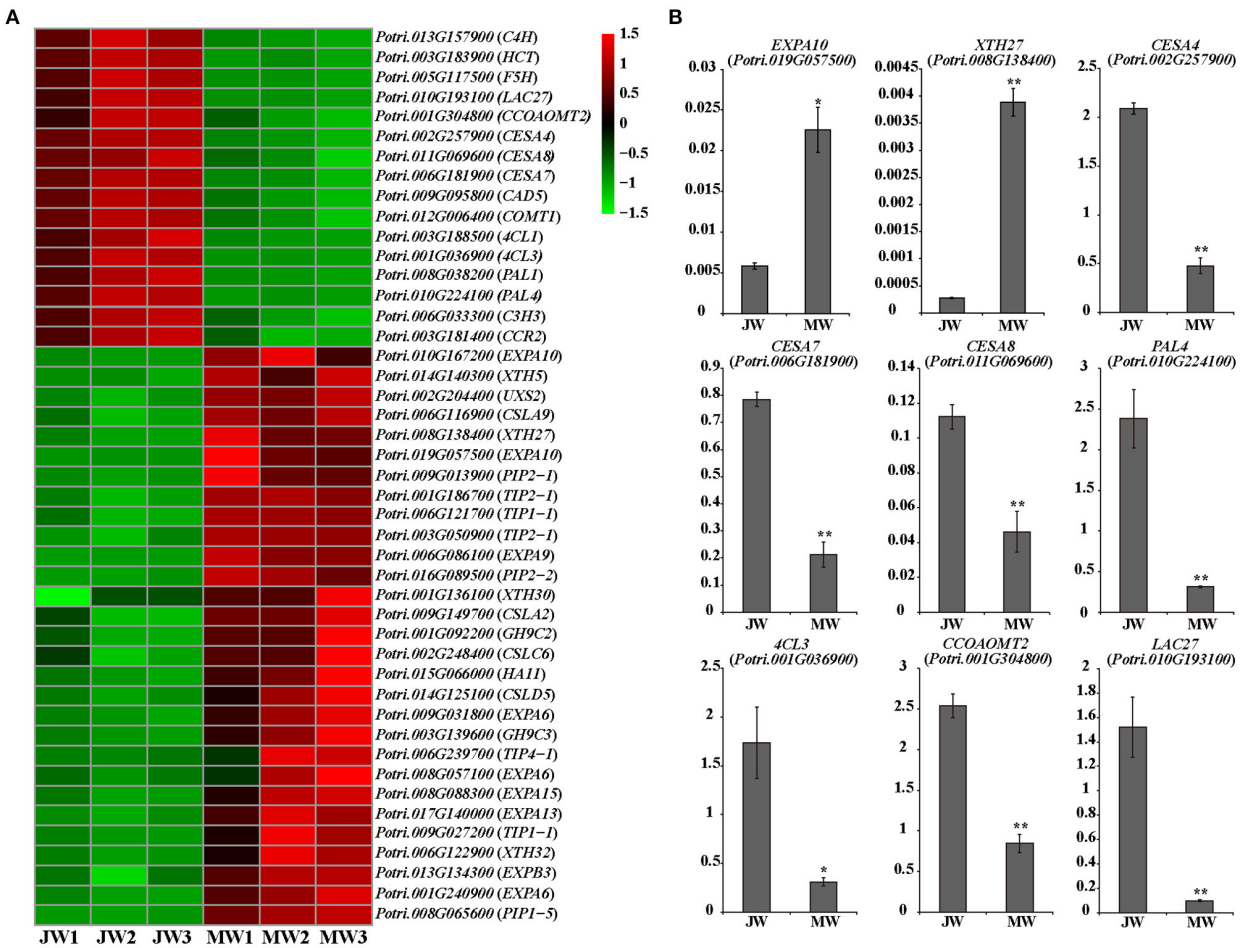
Differentially expressed genes involved in cell wall formation in JW and MW. **(A)** The heat map of genes related to cell wall formation. **(B)** The results of quantitative real-time PCR of selected genes related to cell wall formation in JW and MW. Significance was determined by Student's *t*-test (*p < 0.05 and **p < 0.01).

The DEGs included a large number of genes responsible for cell wall biosynthesis (Figure 4; Supplementary Table 6). It is worth noting that among the DEGs, a large number of genes related to turgor maintenance and cell expansion were identified, of which the majority were upregulated in MW (Figure 4A; Supplementary Table 6). Cell turgor pressure is closely related to cell expansion, it can induce irreversible cell expansion (Genard et al., 2001). Five *TIPs* and three *PIPs* which are related to maintenance of turgor pressure were upregulated in MW. On the other hand, nine *EXPAs* and four *XTHs* which were related to cell wall loosening (Mcqueenmason et al., 1992; Van Sandt et al., 2007; Nishikubo et al., 2011) were identified upregulated in MW (Figure 4A). In addition, the homologs of *FUC1, PAEs, PMEs*, and *PMRs*, which play a role in modifying cell wall for cell wall loosening (Gou et al., 2012; Kato et al., 2018), were also upregulated in MW. Meanwhile, the homolog of *HA11* (a PM H+-ATPase) were upregulated in MW. The PM H+-ATPase can reduce the pH in the apoplast space to activate expansins and other cell wall loosening proteins as well as promote the absorption of water to provide turgor pressure for cell expansion (Spartz et al., 2014). These changes of the gene expression were confirmed by qRT-PCR (Figure 4B). These results support the observation that MW is formed with larger and longer fibers and vessel elements.

Expression of the genes for monolignol biosynthesis was consistently lower in MW, including five *PAL* homologs, *C4H1* and *C4H2, 4CL1* and *4CL5, HCT1, C3H3, CSE2, CCoAOMT1* and *CCoAOMT2, CCR2, F5H1* and *F5H2, COMT1*, and *CAD* (Figure 4A). This may reflect less lignin biosynthesis in MW. After biosynthesis, monolignols are polymerized by laccases or peroxidases. Among detected, 11 *laccase* (*LAC*) genes including homologs of *LAC2, LAC10, LAC11*, and *LAC17* were downregulated in MW. Interestingly, among the 10 detected *peroxidase* genes, two *peroxidase*s were expressed higher in JW and the other eight were expressed higher in MW. It is worthy of further investigating whether the different members of *LAC* or *PRX* act in different phases of wood formation. Cellulose is synthesized by cellulose synthase complex (CSC) of synthases (CesAs) (Song et al., 2010; McFarlane et al., 2014). It is interesting to notice that the homologs of *CesA4, CesA7*, and *CesA8*, which form CSCs for cell wall thickening (Song et al., 2010; Watanabe et al., 2015; Xi et al., 2017), were downregulated in MW (Figures 4A,B). As cellulose synthesis is affected by CesA modifications at protein level (Polko and Kieber, 2019), it is unclear whether modification of CesAs are involved in regulating cellulose synthesis in JW and MW.

A number of genes for biosynthesis of hemicelluloses were differentially expressed in JW and MW (Figure 4A). UDP-glucuronic acid decarboxylase (UXS) catalyzes UDP-glucuronic acid (UDP-GlcA) to biosynthesis of UDP-Xyl (Kuang et al., 2016), which is a donor for biosynthesis of xylan, a major secondary cell wall hemicellulose. *Cellulose synthase-like D* (*CSLD*) involved xylan synthesis (Bernal et al., 2007). *Cellulose synthase-like A* (*CSLA*) encodes mannan synthase (Liepman et al., 2005; Suzuki et al., 2006; Verhertbruggen et al., 2011). Homologs of *UXS, CSLC, CSLD*, and *CLSA* showed higher expression in MW. The results support a higher content of xylan and mannan deposited in MW than in JW (Table 1).

### DNA Methylation in Formation of JW and MW

**Figure 5:**
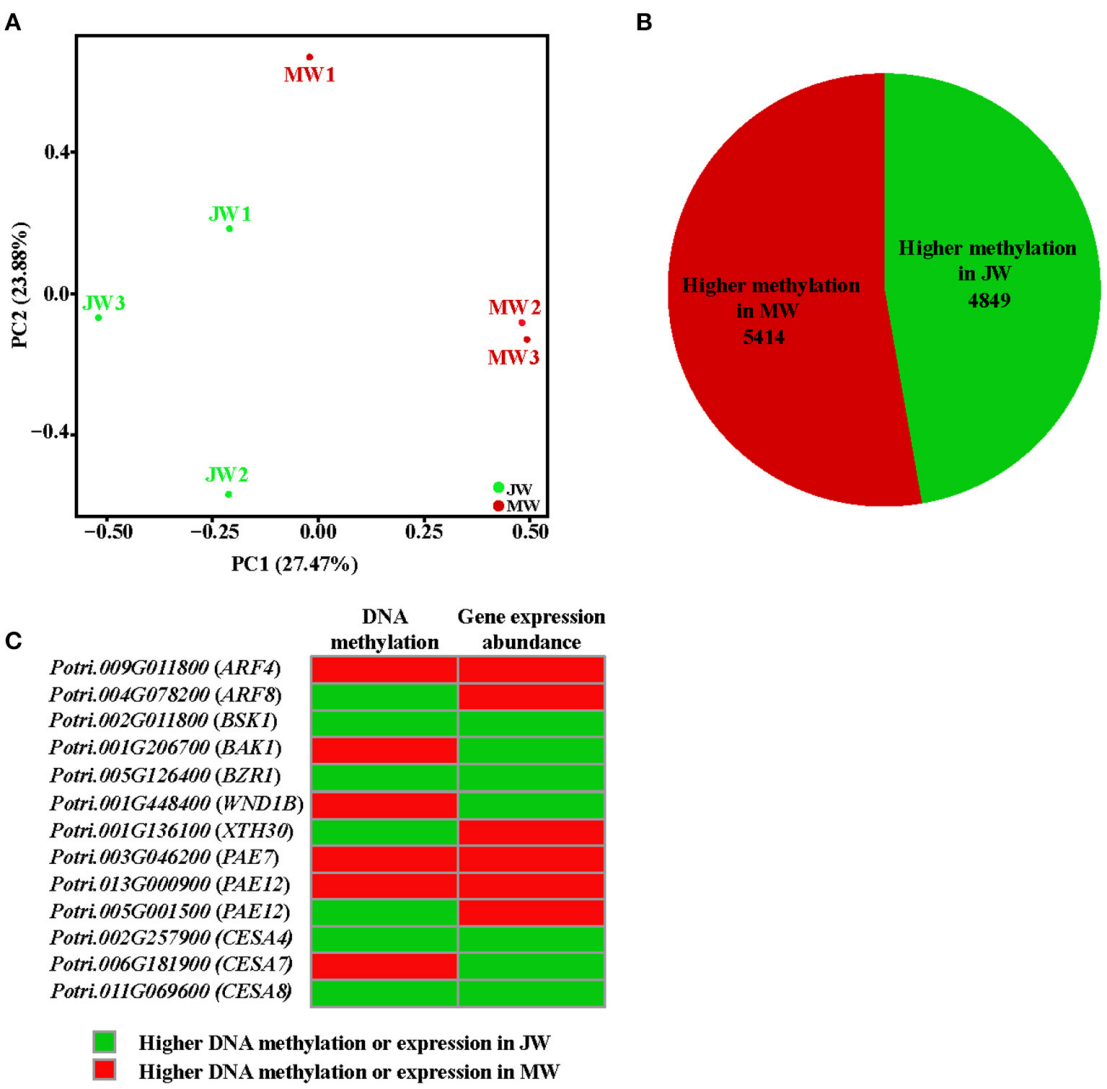
The methylation profile of JW and MW. **(A)** Principal component analysis (PCA) of DNA methylation in each sample. **(B)** The number of higher methylation genes in DMGs. **(C)** Methylation and expression of genes related to wood formation.

The differential gene expression in different growth phases prompted us to examine the whole genome bisulfite sequencing (WGBS). The bisulfite sequencing showed that 87.6–91.9% of the reads was qualified for methylation assay against the *Populus* genome (http://phytozome.jgi.doe.gov/) (Supplementary Table 7, The raw data in Sequence Read Archive (SRA), ID: PRJNA705570). Overall, the methylation level was different within cytosine methylation contexts (CG, CHG, and CHH). The context of CG had higher methylation level, while CHG and CHH had lower (Supplementary Table 8). The DNA methylation context patterns displayed a similarity with those previously observed in *Populus* (Vining et al., 2012; Su et al., 2018). PCA showed that JW and MW had distinct DNA methylation (Figure 5A). Comparison of the DNA methylation in JW and MW revealed 12,176 differentially methylated regions (DMRs) (with methylation difference ≥10, *Q*-value < 0.05). Majority of DMRs were in the contexts of CG sites (10,303) and CHG sites (1,663) (Supplementary Figure 4A; Supplementary Table 9), Among them, 10,237 DMRs were located in gene body and/or flanking regions (±2 kb), named differentially methylated genes (DMGs). In MW, 5,414 DMGs showed higher methylation while JW contained 4,849 DMGs with higher methylation (Figure 5B; Supplementary Table 10), suggesting that different DNA methylations occurred in the formation of JW and MW. Analysis of the correlation between DMGs and DEGs indicated that DMRs in gene promoter region were more likely to affect gene expression (Supplementary Figure 4B). About 20% DEGs (802) displayed different methylation (Supplementary Table 11). These DEGs were closely related to plant hormone signaling and response, cell wall formation and modification, metabolic process, transcription and translation, etc. (Supplementary Figure 4C; Supplementary Table 12). For example, the homologs of *ARFs, BAK1, BSK1*, and *BZR1*, which are involved in auxin and BR signaling, were differential methylated in their different gene regions in JW and MW (Figure 5C; Supplementary Table 11). Furthermore, several genes related to cell wall formation such as *XTH30, PAEs, WND1B, CESA4, CESA7*, and *CESA8* (Figure 5C; Supplementary Table 11) showed differential methylation in JW and MW. In addition, several DMRs in intergenic region were neighbored to the homologs of *PILS2, AUX1, PIN7, WND2A, MYBs*, and *PAL1*, which are involved in auxin distribution and cell wall biosynthesis (Supplementary Table 11). In summary, the results revealed that DNA methylation displayed a clear difference in the formation of JW and MW, which may play a role in regulating gene expression in different growth phases, particularly for the genes involved in hormone signal transduction, cell division, and cell wall biosynthesis in wood formation.

## Discussion

At a given point of tree development, wood can be differentiated into juvenile wood and mature wood which have distinct properties (Basheer-Salimia, 2007; Barrios et al., 2017). In the present study, we profiled the transcriptome and DNA methylation patterns in JW and MW derived from an identical genetic background in order to uncover the paths involved in wood formation at different developmental phases. Different transcription profiles and DNA methylation were identified in the formation of JW and MW. Differences in gene expression were primarily associated with plant hormones including auxin and BR signaling and response, cell wall formation and modification, cell organization and biogenesis, and transcription regulation processes. Different patterns of DNA methylation were also detected in genes involved in auxin transport, BR signaling, and cell expansion which suggest a role for the epigenetic regulation of JW and MW formation.

Different expressions of auxin transport genes were observed in JW and MW. Relative to JW, we observed that the genes related to auxin influx (homolog of *AUX1*) (Enders and Strader, 2015) were downregulated in MW, while the genes related to auxin efflux (homologs of *PINs, PILSs, ABCB19*) (Liu et al., 2014; Enders and Strader, 2015) were upregulated in MW. Meanwhile, the different members of the *AUX1/LAX3* family were that expressed in JW and MW imply a possibility that formation of JW and MW involves distinct auxin molecule formats, as *AUX1/LAX* members correspond with different auxin formats (Enders and Strader, 2015). Further characterization of PIN, ABCB, AUX1, and LAX3 proteins in association with auxin in the JW-/MW-forming tissues would be able to provide mechanistic evidence for verification of the findings. However, current results indicate that auxin transport plays a role in regulating the formation of JW and MW.

Furthermore, we also found that homolog of *DIM* which is a key gene for BR biosynthesis (Klahre et al., 1998) was upregulated in MW, while homologs of *BAK1, BSK1*, and *BZR1* which are marker genes for BR signaling (Li et al., 2002; Nam and Li, 2002; Wang et al., 2002; Tang et al., 2008) showed downregulated expression in MW, suggesting that BR signaling plays a role in regulating MW formation. Studies have shown that BR promotes wood formation (Du et al., 2020). It is worthy of studying whether BR manipulates wood properties in wood formation because the properties of JW and MW are different.

DNA methylation acts as an epigenetic mechanism to regulate gene expression in plants (Fraga et al., 2002a; Vining et al., 2012; Matzke and Mosher, 2014; Liang et al., 2019). In this study, we found that the methylation level of the auxin transport genes *PILS2, AUX1*, and *PIN7* was different between JW and MW. In addition, the different degrees of DNA methylation were also detected in the BR signaling genes *BAK1, BSK1*, and *BZR1*. It is likely that the different expressions of these genes in JW and MW may be related to their DNA methylation changes over the developmental process. Further investigation of the DNA methylation effect on the transcription activities of the auxin and BR genes would help in the revelation of the molecular pathways underlying the alternation of the hormone signaling during different development phases in perennial trees. In summary, the present results suggest that auxin distribution and transportation, BR biosynthesis, and signaling are involved in regulating the wood formation at juvenile and mature phase. DNA methylation plays an important role in regulating the expression of the auxin and BR genes at different development phases.

In consistent with the hormone signaling changes, the downstream biological processes in response to auxin and BR also showed alternation in JW and MW. For instance, TFs such as *ARFs, SRSs, AP2*, and *MYB3R1* and genes related to cell loosing and cell expansion such as *HA11, XTHs, FUC1, PAEs, PMEs, PMRs, PIPs*, and *TIPs*, of which the expression is responding to auxin signaling (Guilfoyle and Hagen, 2007; Spartz et al., 2014), showed differential expression in formation of JW and MW. These transcription regulations are in agreement with the MW properties that have significantly longer and larger fiber cells and vessels.

Cell wall composition (including lignin, cellulose, and hemicellulose) which is closely related to wood properties is rather different in JW and MW (Table 1). Expressions of the genes related to lignin biosynthesis were downregulated, and the genes for hemicelluloses biosynthesis were upregulated in MW, consistent with the result of less lignin content and higher hemicellulose content in MW. Interestingly, expression of the cellulose biosynthesis genes (such as *CesA4, CesA7*, and *CesA8*) was downregulated in MW compared with JW. However, the cellulose content was higher in MW. As this discrepancy requires further verification, regulation of the CesA activity at protein level may be considered. It is known that protein phosphorylation plays a crucial role in regulating CesA catalytic activity and motility (Chen et al., 2010; Speicher et al., 2018; Polko and Kieber, 2019). More evidence is needed for the elucidation of the different cellulose accumulations in JW and MW.

## Conclusions

**Figure 6:**
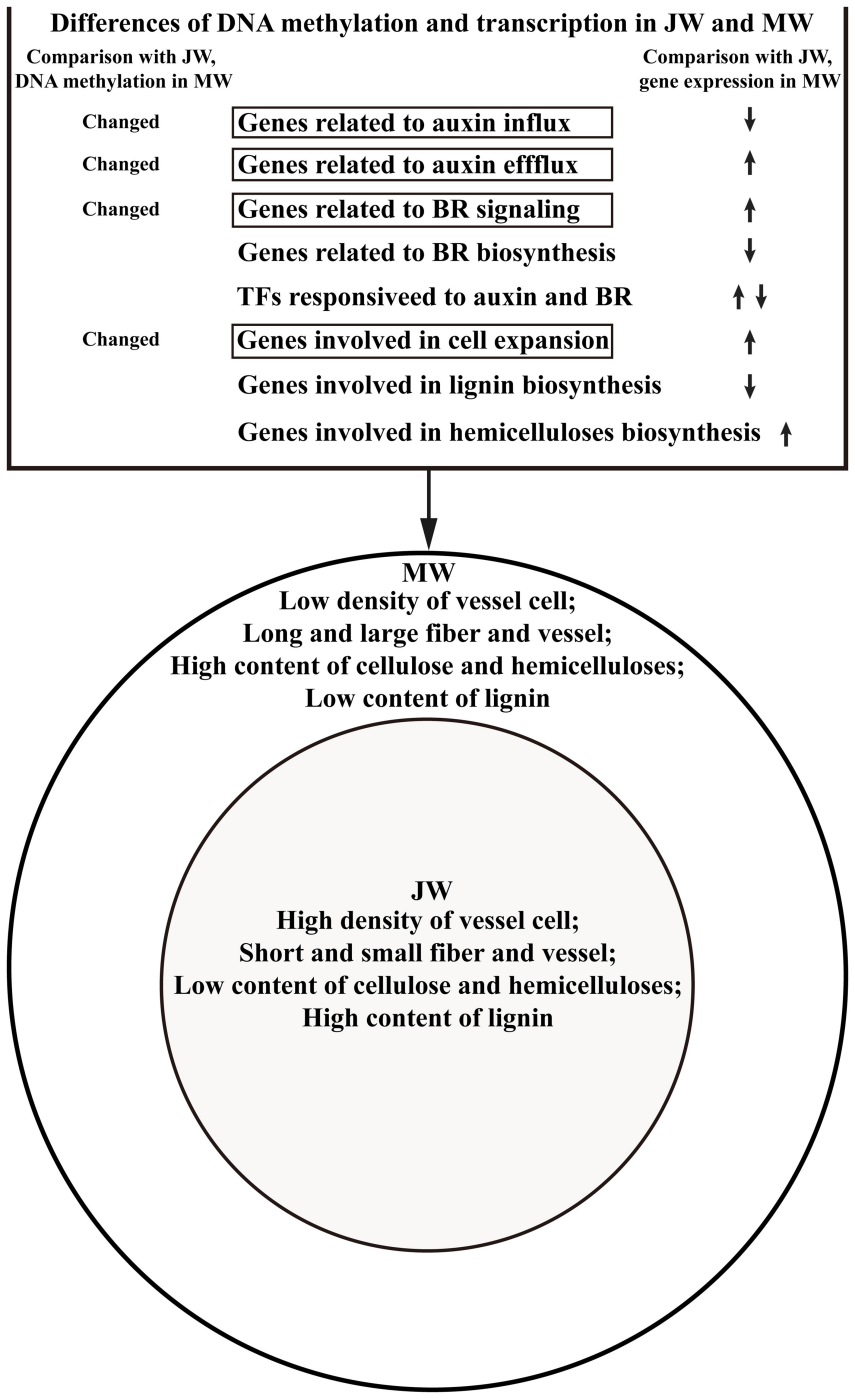
Outline of the DAN methylation and transcription regulation in formation of JW and MW.

In this study, we analyzed transcription profiles and genome-wide DNA methylation in association with the wood properties of JW and MW by employing *Populus* trees with an identical genetic background. Results suggest that auxin distribution and BR signaling may act as major mechanisms to modulate the wood formation in different development phases. In response to the hormone signaling alteration, the transcription activities are modulated, leading to the formation of different wood properties in JW and MW. Furthermore, results also indicate that the transcription modulation of the hormone-related genes may be regulated through DNA methylation. The study outlines a picture of the main transcription networks related to wood formation in JW and MW and a possible role of DNA methylation in tuning the transcriptional network (Figure 6). These findings shed light toward a better mechanistic understanding of wood formation in different development phases and new evidence to inform the engineering of wood properties.

## Data Availability Statement

The data presented in the study are deposited in Sequence Read Archive (SRA) repository, the RNA-seq accession number: PRJNA705066 and DNA methylation accession number: PRJNA705570). Supporting results of this article are included in Supplementary Material.

## Author Contributions

LLu performed experiments, analyzed data, and wrote the manuscript. YZ and JL analyzed data and wrote the manuscript. JG conducted RNA-seq analysis and wrote the manuscript. TY prepared the tree samples and analyzed data. LLi conceived the project, analyzed data, and wrote the manuscript. All authors have read and approved the final manuscript.

## Conflict of Interest

The authors declare that the research was conducted in the absence of any commercial or financial relationships that could be construed as a potential conflict of interest.
