# Supplementary material for: A Comparative Analysis of Transcription Networks Active in Juvenile and Mature Wood in Populus
Source: Front Plant Sci. 2021 May 28;12:675075. doi: 10.3389/fpls.2021.675075 (PMC8193101; doi:10.3389/fpls.2021.675075)
Supplement: Supplementary file 1 [file Table_1.DOC]

**SUPPLEMENTARY TABLE S1| Quality assessment of the RNA-seq data from JW and MW.**

| Sample | JW1 | JW2 | JW3 | MW1 | MW2 | MW3 |
| --- | --- | --- | --- | --- | --- | --- |
| Raw reads | 50173624 | 50684990 | 49424646 | 50681358 | 49717598 | 49833570 |
| Raw bases | 7526043600 | 7602748500 | 7413696900 | 7602203700 | 7457639700 | 7475035500 |
| Valid bases (%) | 94.57 | 94.89 | 95.19 | 94.65 | 95.06 | 93.66 |
| Clean reads | 47498330 | 48145086 | 47096164 | 48018094 | 47306200 | 46722242 |
| Total mapped (%) | 41426989 (87.22) | 41641336 (86.49) | 40818802 (86.67) | 41638789 (86.71) | 40936483 (86.54) | 40345648 (86.35) |
| Multiple mapped (%) | 1344595 (2.83) | 1366306 (2.84) | 1354159 (2.88) | 1364038 (2.84) | 1324686 (2.79) | 1294844 (2.77) |
| Unique mapping (%) | 40082394 (84.39) | 40275030 (83.65) | 39464643 (83.79) | 40274751 (83.87) | 39611797 (83.73) | 39050804 (83.58) |
| Read-1 (%) | 20075500 (42.27) | 20158229 (41.87) | 19751020 (41.94) | 20160072 (41.98) | 19809744 (41.88) | 19595842 (41.94) |
| Read-2 (%) | 20006894 (42.12) | 20116801 (41.78) | 19713623 (41.86) | 20114679 (41.89) | 19802053 (41.86) | 19454962 (41.64) |
| Reads map to '+' (%) | 20013054 (42.13) | 20107035 (41.76) | 19706254 (41.84) | 20115319 (41.89) | 19793522 (41.84) | 19505768 (41.75) |
| Reads map to '-' (%) | 20069340 (42.25) | 20167995 (41.89) | 19758389 (41.95) | 20159432 (41.98) | 19818275 (41.89) | 19545036 (41.83) |
| Unigenes | 20119 | 19676 | 19871 | 20083 | 20338 | 20456 |

Raw reads: number of raw reads; Raw bases: number of raw bases; Valid bases: the percentage of valid bases; Clean reads: number of clean reads after filtering; Total mapped: clean reads matched on the genome; Multiple mapped: number of clean reads with multiple aligned positions; Unique mapping: number of clean reads with unique aligned position; Read-1 / Read-2: reads on the reference genome for left reads / right reads, respectively; Reads map to '+' / Reads map to '-': sequence alignment on positive and negative strands on the genome; Unigenes: number of mapped genes.
