# Supplementary material for: A Comparative Analysis of Transcription Networks Active in Juvenile and Mature Wood in Populus
Source: Front Plant Sci. 2021 May 28;12:675075. doi: 10.3389/fpls.2021.675075 (PMC8193101; doi:10.3389/fpls.2021.675075)
Supplement: Supplementary file 7 [file Table_7.DOC]

**SUPPLEMENTARY TABLE S7| Quality assessment of the BS-seq data from JW and MW.**

|  | JW1 | JW2 | JW3 | MW1 | MW2 | MW3 |
| --- | --- | --- | --- | --- | --- | --- |
| Raw reads | 150302452 | 148753216 | 151839958 | 135565332 | 140475928 | 143338966 |
| Clean reads (%) | 134330976 (89.37) | 130322286 (87.61) | 133650426 (88.02) | 122624418 (90.45) | 129110746 (91.91) | 130059480 (90.74) |
| Uniquely mapped | 32840936 | 25505956 | 28963288 | 25848968 | 26714966 | 27056676 |
| Multiple mapped | 9354446 | 7527320 | 9195070 | 5847736 | 7530414 | 7660828 |
| Unmapped | 92135594 | 97289010 | 95492068 | 90927714 | 94865366 | 95341976 |
| Mapping rate (%) | 31.41 | 25.35 | 28.55 | 25.85 | 26.52 | 26.69 |
| Conversion rate (%) | 99.55 | 99.43 | 99.43 | 99.78 | 99.28 | 99.59 |
| Genes coverage (%) | 87.52 | 85.67 | 90.83 | 81.86 | 83.77 | 84.96 |
| Raw reads: number of raw reads; Clean reads: number of clean reads after filtering; Uniquely mapped: number of clean reads with unique aligned position; Multiple mapped: number of clean reads with multiple aligned positions; Unmapped: number of clean reads with not mapped to the reference genome; Mapping rate: (Uniquely mapped+ Multiple mapped) / clean reads; Conversion rate: converted cytosine sites/total cytosine sites in landDNA after bisulfite treated; Genes coverage: ratio of detected genes/total genes. | | | | | | |
