# Supplementary material for: A Comparative Analysis of Transcription Networks Active in Juvenile and Mature Wood in Populus
Source: Front Plant Sci. 2021 May 28;12:675075. doi: 10.3389/fpls.2021.675075 (PMC8193101; doi:10.3389/fpls.2021.675075)
Supplement: Supplementary file 8 [file Table_8.DOC]

**SUPPLEMENTARY TABLE S8| The methylation ratio in different sequence contexts (CG, CHG, and CHH).**

|  | Context | Sample | Covered C | mC | mC percent (%) | Ave (%) |
| --- | --- | --- | --- | --- | --- | --- |
| JW | C | JW1 | 14414751 | 2982486 | 20.69 | 27.07 |
|  |  | JW2 | 8476604 | 2925969 | 34.52 |  |
|  |  | JW3 | 13929850 | 3621695 | 25.99 |  |
|  | CG | JW1 | 1196046 | 544153 | 45.49 | 51.05 |
|  |  | JW2 | 722263 | 419127 | 58.03 |  |
|  |  | JW3 | 1208547 | 599957 | 49.64 |  |
|  | CHG | JW1 | 2643317 | 526371 | 19.91 | 25.89 |
|  |  | JW2 | 1438724 | 478194 | 33.24 |  |
|  |  | JW3 | 2515382 | 616520 | 24.51 |  |
|  | CHH | JW1 | 10575388 | 1911962 | 18.08 | 24.59 |
|  |  | JW2 | 6315617 | 2028648 | 32.12 |  |
|  |  | JW3 | 10205921 | 2405218 | 23.57 |  |
| MW | C | MW1 | 6113628 | 1720011 | 28.13 | 29.61 |
|  |  | MW2 | 6515685 | 2072634 | 31.81 |  |
|  |  | MW3 | 7389642 | 2133903 | 28.88 |  |
|  | CG | MW1 | 522676 | 281044 | 53.77 | 56.06 |
|  |  | MW2 | 569917 | 331965 | 58.25 |  |
|  |  | MW3 | 634571 | 356402 | 56.16 |  |
|  | CHG | MW1 | 1073832 | 296902 | 27.65 | 29.8 |
|  |  | MW2 | 1124252 | 363856 | 32.36 |  |
|  |  | MW3 | 1283202 | 377148 | 29.39 |  |
|  | CHH | MW1 | 4517120 | 1142065 | 25.28 | 26.48 |
|  |  | MW2 | 4821516 | 1376813 | 28.56 |  |
|  |  | MW3 | 5471869 | 1400353 | 25.59 |  |

Context: the type of methylation site; Covered C: the total of the identified Cytosine sites; mC: number of cytosine sites where methylation occurs; mC percent (%): the proportion of methylated C sites (mC / Covered C); Ave (%): average of mC percent.
