# Supplementary material for: A Comparative Analysis of Transcription Networks Active in Juvenile and Mature Wood in Populus
Source: Front Plant Sci. 2021 May 28;12:675075. doi: 10.3389/fpls.2021.675075 (PMC8193101; doi:10.3389/fpls.2021.675075)
Supplement: Supplementary file 13 [file Table_13.DOC]

**SUPPLEMENTARY TABLE S13| List of primers used in this study.**

| **Gene name** | **Gene ID** | **Forward primer** | **Reverse primer** |
| --- | --- | --- | --- |
| *AUX1* | *Potri.016G113600* | ACCATTCCAACGCCTTTGCTCTC | ATGAGGATAACCGCAGCATCGC |
| *LAX3* | *Potri.002G087000* | ACACATGATCACCTTCTCTTCAGC | CACCCAACCTCCAAGAAATGGG |
| *PIN3b* | *Potri.008G129400* | GCGCACTCATTAGGAGCCAAAG | TCTCTCCCTGTAAGACTGGCCTTC |
| *PIN6a* | *Potri.005G187500* | GCAACAATGGCAATGGCAATCCG | TCCAACAGCGACCGAAGTTGTC |
| *PILS2-1* | *Potri.004G093200* | AGAGAGGAGGGAGGTGAAAGTAGC | CACCCTAGGTTCTGCCAAACAC |
| *DIM* | *Potri.008G084800* | TATTATTCGCCCGGACCTGTGC | CCAGTCCTCCATTCTACGAACAGC |
| *BAK1* | *Potri.001G206700* | AGGAATGGCAGAAAGAGGAGGTG | AGTGTGTGCGTGATGAGTGTGG |
| *BSK1* | *Potri.002G011800* | CGGCGTAGTCTTTGCCATCTAC | AGACACATTGTGCTTGCATCGC |
| *KAO1* | *Potri.014G179100* | ACAGACAACTGTGTGGCTAGAATC | ATGCATGCCCTTTCTCTTGCTTC |
| *BZR1* | *Potri.005G126400* | TGGAAAGGTCCATGGTCAAGCC | CAAGCTACTTGTTGCTACGTTCCC |
| *ARF4* | *Potri.009G011800* | TCTGCTGCACCGGCTTTATGTG | AGTTTGCAGCCAGTAGCATTCCC |
| *ARF8* | *Potri.004G078200* | TTGGGAGAGCAGGGTGTAGAAC | TAAGCCACACCACCACTCATCC |
| *XTH27* | *Potri.008G138400* | *TTGGATGTGACGAGTGGCGAAG* | *GTGAACCAAGGCCAACATTCTGC* |
| *EXPA10* | *Potri.019G057500* | ACAAACGTTGCTGGAGCAGGAG | TTGCCACCCTGTCTTAGAACCC |
| *CESA4* | *Potri.002G257900* | GGGAGTGGAGTGGTCATTGTATGG | TTGCGTGCATATGCTTGCTTGC |
| *CESA7* | *Potri.006G181900* | TCTCCTTGCTATGGGTCCGAATC | ATTGCTTGGTGTCAGGTCCCTTG |
| *CESA8* | *Potri.011G069600* | ACACCTTGGCTGGTGAAACCTG | ACTGGGAAACTTGTTGGTGGTAGC |
| *PAL4* | *Potri.010G224100* | AGGGAAAGGAATTCAACCACTTCG | CTAGCACTCTCCACTTCTTTAGGC |
| *4CL3* | *Potri.001G036900* | AGATGAGGATGCAGGAGAAGTTCC | TTCATCTTCGGTGGCCTGAGAC |
| *CCoAOMT2* | *Potri.001G304800* | TGCGCCGATGAGGAAGTATGTG | GGTCAGCTGCAAGTGCCTTATTG |
| *LAC27* | *Potri.010G193100* | ACAATGGTAAGGGCCCAAATGAG | AGCACGTTGGAAGGTCACTAGG |
| *WND5A* | *Potri.007G014400* | TTCCTCAGTTAGAGAGCCCATCCC | CAGTCACCTTCTGGGTGTTGTTG |
| *MYB170* | *Potri.005G001600* | AGCAACACCAATTTCTACGAGAGC | CGCCGGATTTCCCACAATTAGC |
| *MYB002* | *Potri.001G258700* | TGCCAATGAACATGGATCCGTCAC | GCTTGCATGGAGGTTTCCAACG |
