# Supplementary figures and images for: A Comparative Analysis of Transcription Networks Active in Juvenile and Mature Wood in Populus
Source: Front Plant Sci. 2021 May 28;12:675075. doi: 10.3389/fpls.2021.675075 (PMC8193101; doi:10.3389/fpls.2021.675075)

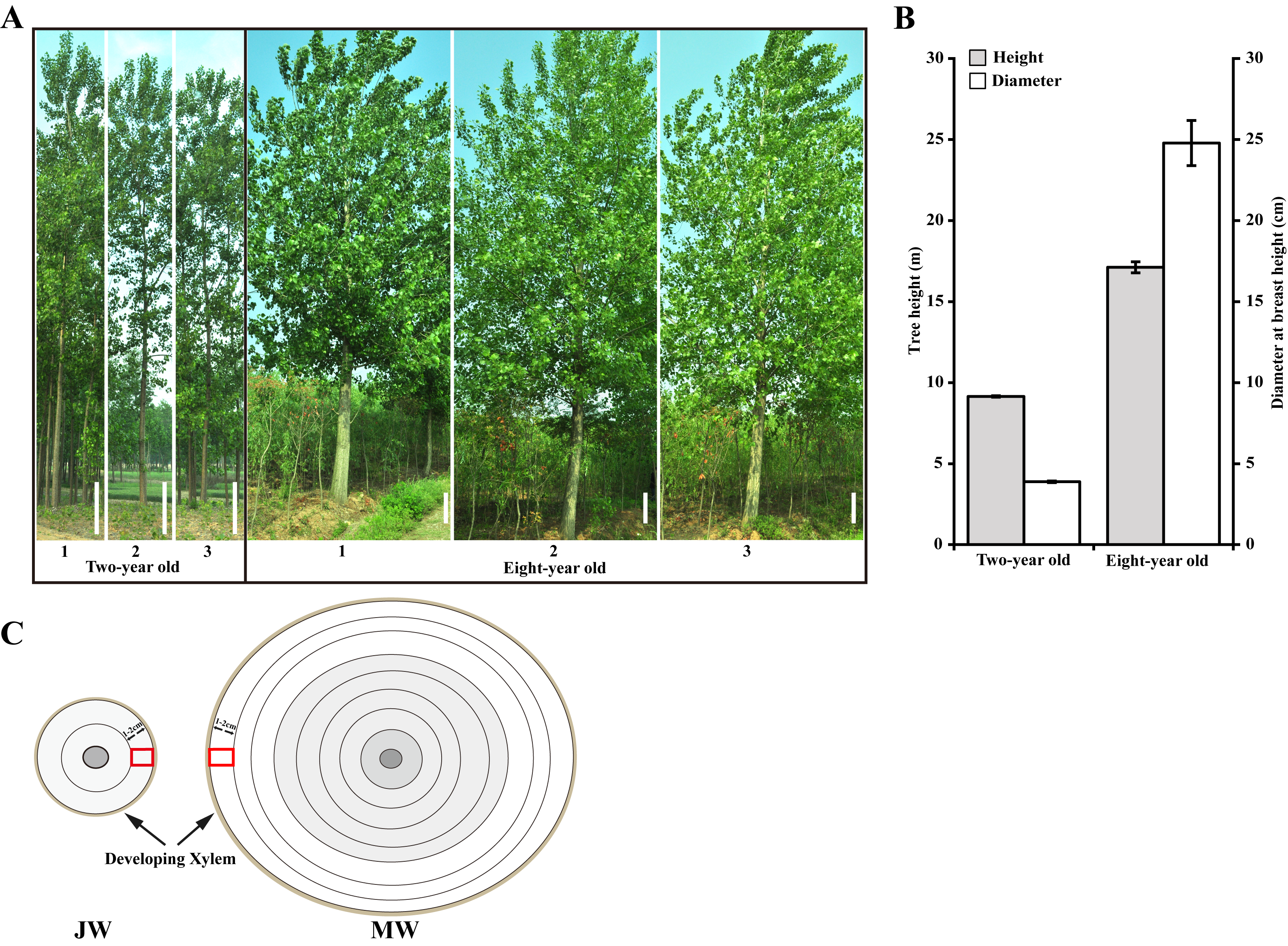

Supplement: Supplementary file 14 [file Image_1.TIF]

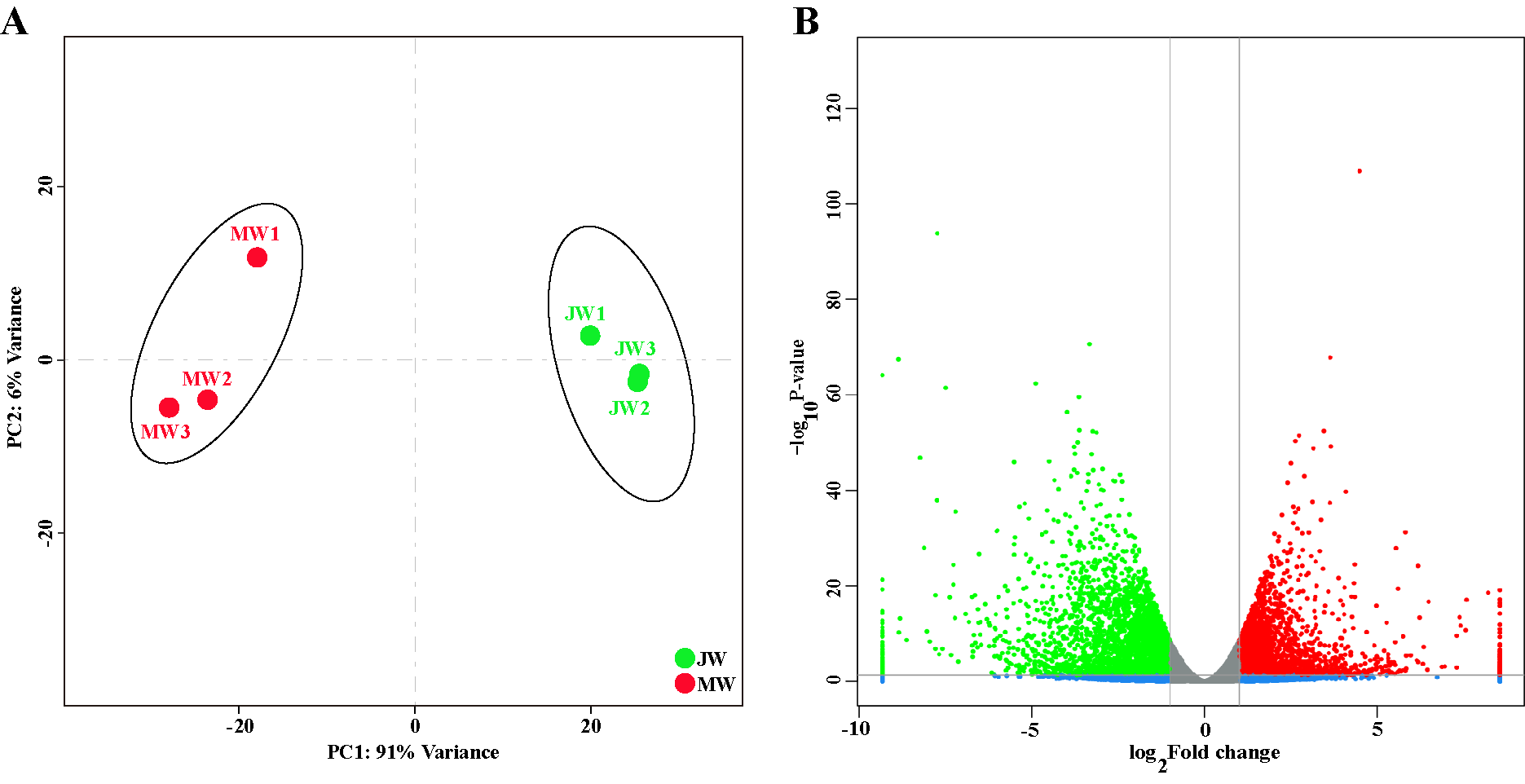

Supplement: Supplementary file 15 [file Image_2.TIF]

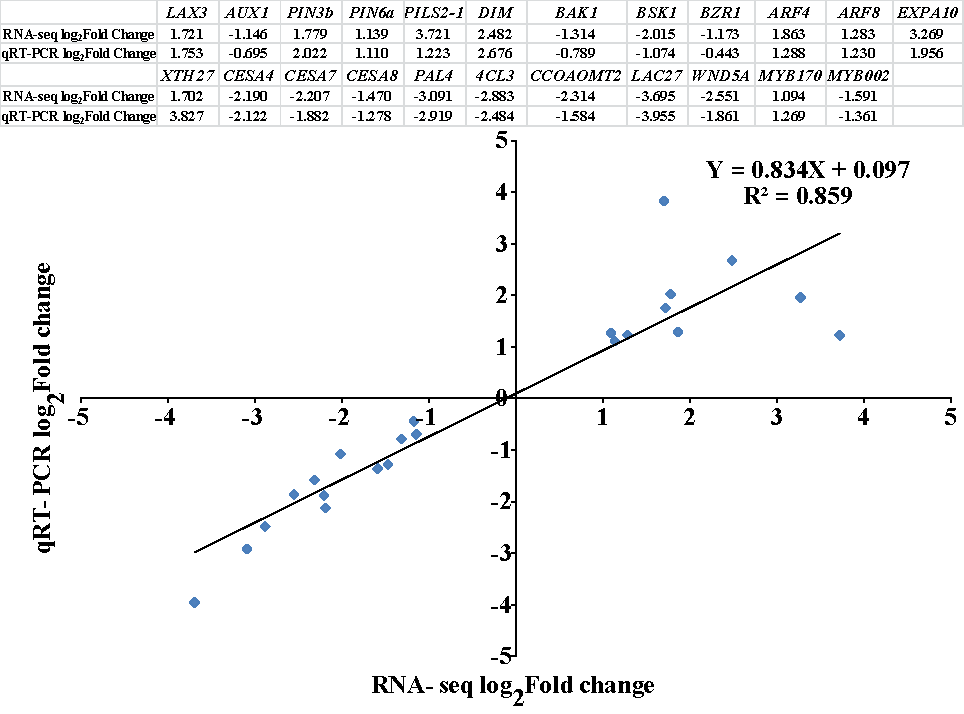

Supplement: Supplementary file 16 [file Image_3.TIF]

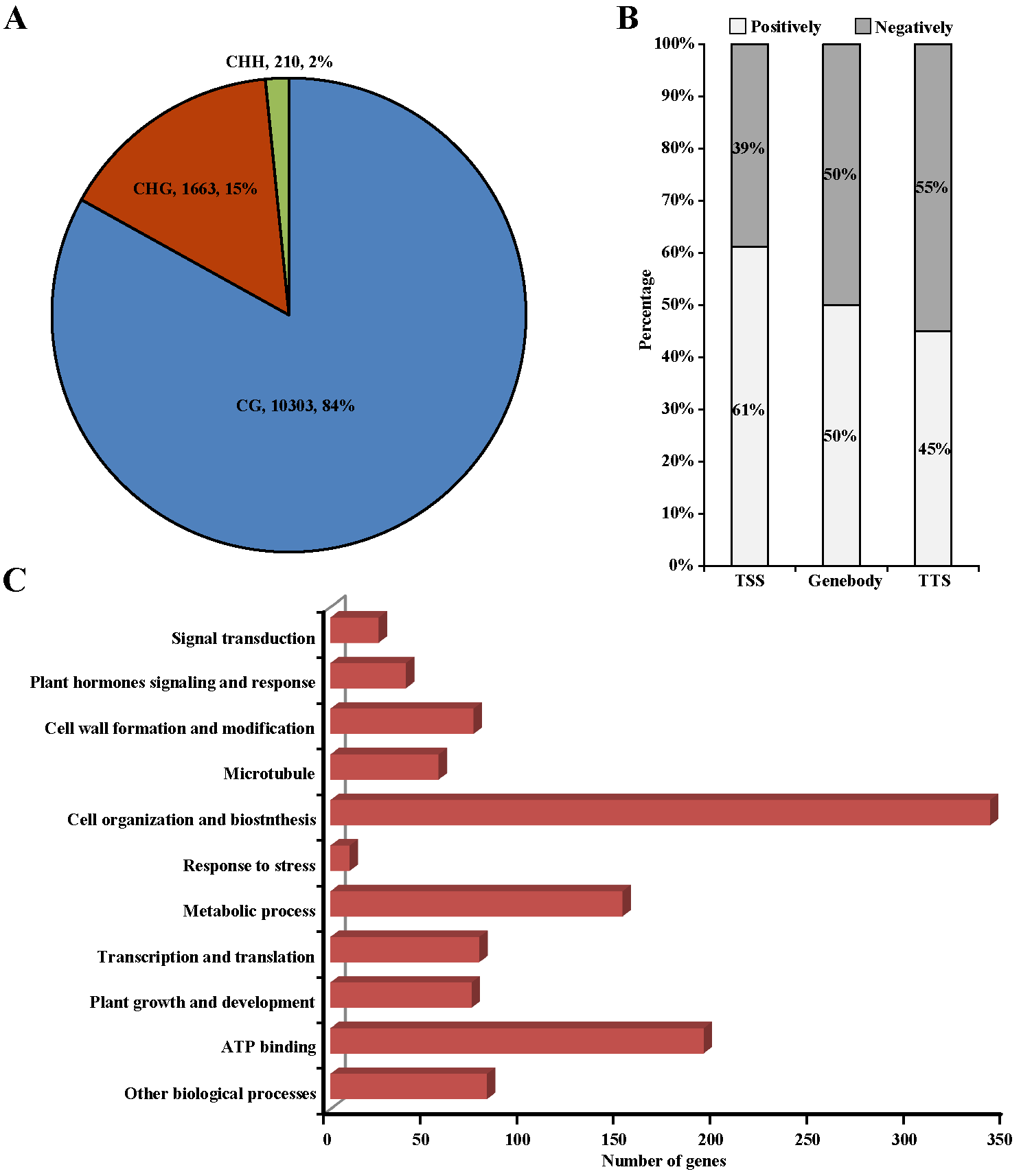

Supplement: Supplementary file 17 [file Image_4.TIF]
