# Supplementary material for: A Comparative Analysis of Transcription Networks Active in Juvenile and Mature Wood in Populus
Source: Front Plant Sci. 2021 May 28;12:675075. doi: 10.3389/fpls.2021.675075 (PMC8193101; doi:10.3389/fpls.2021.675075)
Supplement: Supplementary file 18 [file Data_Sheet_1.docx]

**SUPPLEMENTARY FIGURE AND TABLE LEGENDS:**

**SUPPLEMENTARY FIGURE S1|** **(A)** Trees used for analysis of wood properties and samples of the wood-forming tissues in juvenile and mature phases. Bars=1 meter. **(B)** Tree height and diameter at breast height. **(C)** The illustration of sampling. Arrows indicate developing xylem of the trunk after debarked, which was sampled for RNA-seq and WGBS. Red boxes indicate the wood area used for wood properties analysis.

**SUPPLEMENTARY FIGURE S2|** **(A)** Principal Component Analysis (PCA) of gene expression in each sample. **(B)** Scatter plot of log2 FC versus -log10 p-value of all genes. Green, significantly higher expression (fold change >2 and p-valueFDR ≤ 0.05) in JW; Red, significantly higher expression (fold change >2 and p-valueFDR ≤ 0.05) in MW; Blue, gene expression with fold change >2 and p-valueFDR ≤ 0.05; Gray, genes insignificantly differentially expressed in JW and MW.

**SUPPLEMENTARY FIGURE S3|** The linear correlation between RNA-seq and qRT-PCR.

**SUPPLEMENTARY FIGURE S4| (A)** The distribution of DMRs in different sequence context (CG, CHG and CHH). **(B)** The relationship between DMRs and EDGs in different gene regions. **(C)** Gene ontology (GO) enrichment of genes coexisting in DEGs and DMGs.

**SUPPLEMENTARY TABLE S1|** Quality assessment of the RNA-seq data from JW and MW.

**SUPPLEMENTARY TABLE S2|.** List of differentially expressed genes (DEGs) between JW and MW.

**SUPPLEMENTARY TABLE S3|** Gene ontology (GO) enrichment of the differentially expressed genes (DEGs).

**SUPPLEMENTARY TABLE S4|** KEGG enrichment of the differentially expressed genes (DEGs) in JW and MW.

**SUPPLEMENTARY TABLE S5|** Differentially expressed transcription factor genes.

**SUPPLEMENTARY TABLE S6|** Differentially expressed genes related to cell wall formation.

**SUPPLEMENTARY TABLE S7|** Quality assessment of the BS-seq data from JW and MW.

**SUPPLEMENTARY TABLE S8|** The methylation ratio in different sequence contexts (CG, CHG, and CHH).

**SUPPLEMENTARY TABLE S9|** Differentially methylated regions (DMRs) in JW and MW.

**SUPPLEMENTARY TABLE S10|** Differentially methylated regions (DMGs) in JW and MW.

**SUPPLEMENTARY TABLE S11|** Correlation analysis of DEGs and DMGs.

**SUPPLEMENTARY TABLE S12|** Gene ontology (GO) enrichment of genes which coexist in DEGs and DMGs.

**SUPPLEMENTARY TABLE S13|** List of primers used in this study.
